# Genome-Wide Identification of the *PsatGST* Gene Family and Expression Analysis Under Freezing Stress in Peas (*Pisum sativum* L.)

**DOI:** 10.3390/genes17091052

**Published:** 2026-08-31

**Authors:** Zaoxia Niu, Lijuan Zhang, Bolin Sun, Gengmei Min, Zongwen Chai, Yang Shao

**Affiliations:** 1 Institute of Crop Science, Gansu Academy of Agricultural Sciences, Lanzhou 730070, China; 15719327746@163.com (Z.N.); binglingkeer103@163.com (L.Z.); mingengmei@gsagr.cn (G.M.); 2 Zhangye City Economic Crop Technology Promotion Station, Zhangye 734000, China; 17899314605@163.com; 3 Gansu Provincial General Station of Agricultural Technology Extension, Lanzhou 730020, China; 4 Linxia Hui Autonomous Prefecture Academy of Agricultural Sciences, Linxia 731100, China

**Keywords:** pea, glutathione S-transferase, genome-wide identification, freezing stress, transcriptome, expression analysis

## Abstract

Background: Glutathione S-transferases (GSTs) play pivotal roles in plant growth, abiotic stress responses, detoxification of xenobiotics, and maintenance of redox homeostasis. Methods: In this study, transcriptomic analysis was employed to identify differentially expressed genes (DEGs) in the pea cultivar DX27 under freezing stress (−4 °C) at 3, 6, and 12 h. Results:GO and KEGG enrichment analyses of global transcriptomic data further reveal that DEGs are significantly enriched in the following functional categories: oxidation–reduction processes, stress responses, glutathione metabolism, and secondary metabolite biosynthesis. A total of 52 *PsatGST* genes were identified and classified into nine subfamilies based on phylogenetic relationships. Chromosomal localization revealed a non-random distribution across seven chromosomes. Promoter cis-element analysis indicated that *PsatGST* genes harbor diverse regulatory elements associated with light responsiveness, hormone signaling (auxin, abscisic acid, gibberellin, salicylic acid, and MeJA), and abiotic/biotic stress responses. *PsatGSTF2* expression analysis showed pronounced up-regulation under freezing stress (−4 °C) at 3, 6, and 12 h. Conclusions: These findings provide a foundational framework for understanding the evolutionary history and functional diversification of the *PsatGST* gene family and offer valuable candidate genes for the breeding of stress-tolerant pea varieties.

## 1. Introduction

Glutathione S-transferases (GSTs, EC 2.5.1.18) are a multifunctional superfamily in plants that play important roles in abiotic stress tolerance, detoxification of xenobiotics, and maintenance of cellular redox regulation [1,2]. These enzymes catalyze the conjugation of reduced glutathione (GSH) to electrophilic substrates, thereby facilitating their sequestration into vacuoles or the apoplast [3,4]. Among the various GST classes, the plant-specific Phi (GSTF) and Tau (GSTU) members have been demonstrated to contribute to stress defense through multiple mechanisms [5]; they not only mediate detoxification via GSH conjugation but also directly scavenge reactive oxygen species (ROS) and participate in hormone signaling and stress-responsive kinase regulation [6].

Substantial evidence has shown that *GST* expression and activity are significantly altered in response to various abiotic stresses [7]. For example, plants employ *GSTs* and other antioxidant enzymes to counteract excessive ROS accumulation induced by drought [8,9]. Under freezing stress, elevated ROS levels can damage proteins and compromise membrane integrity [10]. GSTs mitigate such damage by catalyzing the conjugation of GSH with lipid peroxides [2]. Similarly, in herbicide detoxification, GSTs catalyze the nucleophilic attack of GSH on electrophilic groups of hydrophobic compounds, conferring herbicide resistance and selectivity [11,12]. Collectively, these findings underscore the central role GST-mediated glutathione conjugation plays in how plants adapt to environmental stresses [5].

The *GST* gene family has been systematically characterized in several plant species, including *Arabidopsis thaliana* [13], *Oryza sativa* [14], *Glycine max* [15], and *Triticum aestivum* [16]. However, comprehensive genome-wide identification and expression analyses of the GST family under freezing stress have not yet been reported in peas (*Pisum sativum* L.), a legume crop with moderate cold tolerance but limited genomic resources. Previous studies in pea have identified several *GST* genes induced by abiotic stresses, including drought and salinity, but their specific functions remain largely unknown. However, comprehensive genome-wide identification and freezing-responsive expression analysis of the GST family have not yet been reported in pea.

Peas are a cool-season crop well suited to early spring planting in northern China [17]. However, spring frost and freezing stress during seedling establishment remain major constraints on pea productivity and geographical distribution [18]. In this study, we performed the first systematic characterization of the *PsatGST* gene family, including its phylogeny, conserved motifs, chromosomal distribution, promoter elements, and time-dependent expression profiles under freezing stress. Our central hypothesis is that specific *PsatGST* subfamilies particularly the Tau and Phi classes are transcriptionally activated under freezing stress and stress defense in pea plants. This work provides a foundational resource for functional studies and offers candidate genes for breeding cold-tolerant legume varieties.

## 2. Materials and Methods

### 2.1. Plant Materials and Stress Treatment

The pea (*P. sativum* L.) cultivar DX27, provided by Gansu Academy of Agricultural Sciences of Lanzhou China, adapted to the alpine-cool agro-ecological zones of northwest China. Plants exhibit semi-erect growth habit, green cotyledons, and medium-sized seeds. Under laboratory conditions, DX27 seedlings display enhanced tolerance to freezing stress compared with local susceptible controls, making it suitable for investigating molecular mechanisms underlying freezing-stress responses. Seeds were propagated and harvested under consistent field conditions. Uniform, plump seeds were surface-sterilized with 75% (*v*/*v*) ethanol for 30 s and 10% (*v*/*v*) sodium hypochlorite for 10 min, followed by five rinses with sterile distilled water. The seeds were then germinated on moist filter paper at 25 °C in darkness for 3 days. Germinated seedlings were first grown hydroponically in nutrient solution (without solid substrate), and then transplanted into Hoagland nutrient solution and cultivated in an artificial climate chamber under controlled conditions: a 16 h light/8 h dark photoperiod, a light intensity of 200 μmol·m^−2^·s^−1^ (cool white fluorescent lamps), a day/night temperature of 25 °C/18 °C, and a relative humidity of 60–70%. Hoagland nutrient solution containing 5 mM KNO_3_, 2 mM MgSO_4_, 2 mM Ca(NO_3_)_2_, 1 mM KH_2_PO_4_, 0.1 mM Fe-EDTA, 20 µM H_3_BO_3_, 5 µM MnCl_2_, 0.5 µM CuSO_4_, 5 µM ZnSO_4_, and 0.5 µM Na_2_MoO_4_. Transplant the seedlings into nutrient-rich soil when the first true leaf emerges. At the three-leaf seedling stage, healthy and uniform seedlings were selected for freezing stress treatment. The 3 h, 6 h, and 12 h time points were selected based on preliminary physiological assays showing that visible freezing injury first appears at approximately 6 h and becomes severe by 12 h. The treatment group was transferred to a climate chamber set at −4 °C, while the control group (CK) was maintained at 25 °C, under the same light and humidity conditions. Leaf tissues were collected at 3, 6, and 12 h after stress initiation, with three biological replicates per time point (each consisting of leaves pooled from five seedlings). Control samples were collected at 3, 6, and 12 h alongside the treated samples to control for time-dependent transcriptional changes. All freezing-stress treatments were completed within 12 h. A single shared control group was used for all three samples time-points (3 h, 6 h, and 12 h). Since the whole treatment procedure was finished in a short time-frame, circadian rhythm-related effects were negligible in this experiment. All samples were immediately frozen in liquid nitrogen and stored at −80 °C for subsequent RNA extraction and transcriptome sequencing.

### 2.2. RNA Extraction and Transcriptome Sequencing

Total RNA was isolated from pea leaf tissues with the RNAprep Pure Plant Plus Kit (Tiangen Biotech, Beijing, China) in strict accordance with the manufacturer’s instructions. RNA purity and integrity were assessed using 1% agarose gel electrophoresis and a NanoDrop 2000 spectrophotometer (Thermo Fisher Scientific, Waltham, MA, USA). Only RNA samples with an RNA integrity number ≥ 8.0 were retained for downstream library construction. Sequencing libraries were generated using the NEBNext^®^ Ultra™ RNA Library Prep Kit for Illumina^®^ (New England Biolabs, Ipswich, MA, USA). In brief, poly (A)-tailed mRNA was captured from total RNA using oligo (dT) magnetic beads and fragmented into short segments with fragmentation buffer. First-strand cDNA was synthesized using random hexamer primers, followed by second-strand cDNA generation with DNA polymerase I and dNTPs. The double-stranded cDNA was then purified, end-repaired, adenylated at the 3′ ends, and ligated with Illumina sequencing adaptors. The ligated products were enriched via PCR amplification, and the qualified cDNA libraries were sequenced on the Illumina NovaSeq 6000 platform (Illumina, San Diego, CA, USA) by Biomarker Technologies Co., Ltd. (Beijing, China) to produce 150 bp paired-end reads.

### 2.3. Identification and Analysis of Differentially Expressed Genes (DEGs)

Clean reads were aligned to the pea reference genome *(P. sativum* v1a) using HISAT2 (version 2.2.1) [19]. Raw read counts per gene were obtained, and genes with <10 counts in all samples were excluded. Differentially expressed genes (DEGs) between cold-stressed and control groups were identified using DESeq2 (v1.40.2) [20] with |log_2_FC| ≥ 1 and Padj < 0.05 (the Benjamini–Hochberg method). FPKM values from StringTie (version 2.1.7) were used solely for heatmap visualization [21]. For every comparison, gene-level dispersion values were estimated with the default estimate Dispersions function of DESeq2 employing parametric fit. As explicitly stated in the original DESeq2 manuscript, dispersion is calculated by means of shrinkage estimation, which makes experimental designs with two to three replicates common and reasonable. Only samples belonging to that specific comparison were used; three independent DESeq2 objects were constructed to prevent confounding effects caused by cross-time-point information sharing [22]. Non-redundant and shared DEGs across the three time-points were visualized by Venn-diagram analysis. Volcano plots were generated for each comparison to illustrate DEG distributions. DEG overlaps and expression trends were visualized using the TBtools (version 1.120) VennDiagram and bar charts.

### 2.4. GO and KEGG Enrichment Analyses

Functional enrichment analysis based on Gene Ontology (GO) was carried out for DEGs using the R package cluster Profiler (v4.8.3) [23]. All GO terms were categorized into three independent ontologies: Biological Process (BP), Cellular Component (CC), and Molecular Function (MF). Pathway enrichment was implemented using the same tool against the Kyoto Encyclopedia of Genes and Genomes (KEGG) database to assign DEGs to relevant metabolic and signal transduction pathways. A false discovery rate (FDR) < 0.05 was employed as the threshold for identifying significantly enriched GO terms and KEGG pathways. The enrichment results were visualized as bubble plots and bar charts using the ggplot2 R package (v3.4.4) [24].

### 2.5. Genome-Wide Identification, Clustering, and Chromosomal Localization of the Pea GST Gene Family

*GST* gene family members were identified using the *P. sativum* v1a reference genome (Phytozome) as the reference database. The hidden Markov model (HMM) profiles of the conserved GST domains (GST-N: PF02798, GST-C: PF00043) were downloaded from the Pfam database (http://pfam.xfam.org) (accessed on 10 August 2025) [25]. HMMER 3.0 [26] was used to search the pea protein database with HMM profiles (E-value ≤ 1 × 10^−5^). Candidate sequences were required to contain at least one complete GST-N (PF02798) or GST-C (PF00043) domain, as verified by the Pfam scan (E-value ≤ 1 × 10^−5^). Redundant isoforms from alternative splicing were removed by retaining the longest transcript. Sequences with incomplete domains or <50 amino acids were discarded.

Multiple sequence alignment of the PsatGST amino acid sequences was performed using ClustalW [27]. Based on the aligned sequences, a phylogenetic tree was constructed using the Neighbor-Joining (NJ) method implemented in MEGA 7.0 software [28]. The resulting phylogenetic tree was further visualized, edited, and optimized using the online tool Evolview (https://evolgenius.info//evolview-v2/#login) (accessed on 10 August 2025) [29]. The chromosomal start coordinates of each *PsatGST* gene were retrieved from the *P. sativum* v1a genome database, and the physical distribution of these genes across pea chromosomes was graphically mapped using the software package MapChart [30].

### 2.6. Identification of Conserved Motifs and Cis-Acting Elements in the Promoters of the PsatGST Gene Family

The full-length coding sequences of *PsatGST* genes were retrieved, and the 2000-bp promoter regions upstream of the translation initiation site (ATG) were extracted for each gene. Putative cis-acting regulatory elements were predicted and analyzed using the PlantCARE online database (http://bioinformatics.psb.ugent.be/webtools/plantcare/html/) (accessed on 10 August 2025) [31], and corresponding regulatory element diagrams were constructed. Conserved motifs within PsatGST proteins were identified using the online tool MEME (http://meme-suite.org) (accessed on 10 August 2026) [32], with the maximum number of motifs set to 10 and other parameters maintained at their default settings. All motif and gene structure visualizations were carried out using the TBtools (version 1.120) software package [33].

### 2.7. Expression Pattern Analysis of PsatGST Genes Under Freezing Stress

Expression patterns of *PsatGST* genes under freezing stress were visualized using FPKM values and used exclusively for heatmap generation. Expression data were normalized via log_2_ transformation, and a heatmap illustrating *PsatGST* expression at 3 h, 6 h, and 12 h under control and cold-stress conditions was generated using TBtools [34]. The expression profiles of key *PsatGST* genes were further characterized and interpreted by integrating heatmap visualization and differential expression statistics calculated from raw counts with DESeq2. RNA Extraction Kits (TIANGEN Biotech Co., Ltd., DP432, Beijing, China) were employed to extract total RNA from both wild-type (WT) and transgenic plants, ensuring the elimination of genomic DNA contamination. The cDNA synthesis was carried out using the PrimeScript™ RT Master Mix (Vazyme, Nanjing, China) following the provided instructions. Subsequently, the quality and concentration of the obtained cDNA were assessed using an ultra-trace UV-visible spectrophotometer (NanoVueTM Plus, Wilmington, DE, USA) and stored in a standby state. All the primer sequences are listed in Table 1. Quantitative real-time PCR (qRT-PCR) was conducted on a LightCycler^®^ 480 II system (Roche, Basel, Switzerland). Each 20-μL reaction contained 10 μL of enzyme mix, 2 μL of cDNA, 0.8 μL each of forward and reverse primers (10 μmol/L), and 6.4 μL of ddH_2_O. The thermal cycling protocol comprised an initial denaturation at 95 °C for 30 s, followed by 40 cycles of 95 °C for 5 s and 60 °C for 30 s. A melting curve analysis was then performed at 95 °C for 5 s, 60 °C for 1 min, and 95 °C for 15 s. All reactions were run with three independent biological replicates, and each biological replicate included three technical replicates, which were averaged before statistical analysis. Relative transcript levels were calculated using the 2^−ΔΔCT^ method.

### 2.8. Statistical Analysis

All experiments were performed with three biological replicates or three technical replicates for the molecular experiments. Graphs were generated using Tbtools and R packages (R (version 4.5.1)) [34]. The statistical test used (one-way ANOVA followed by Duncan’s multiple range test). Clear statement that error bars represent standard deviation (SD) from biological replicates. Technical replicates were averaged before statistical analysis.

## 3. Results

### 3.1. Identification of Differentially Expressed Genes (DEGs) in Pea Plants Under Freezing Stress

**Figure 1:**
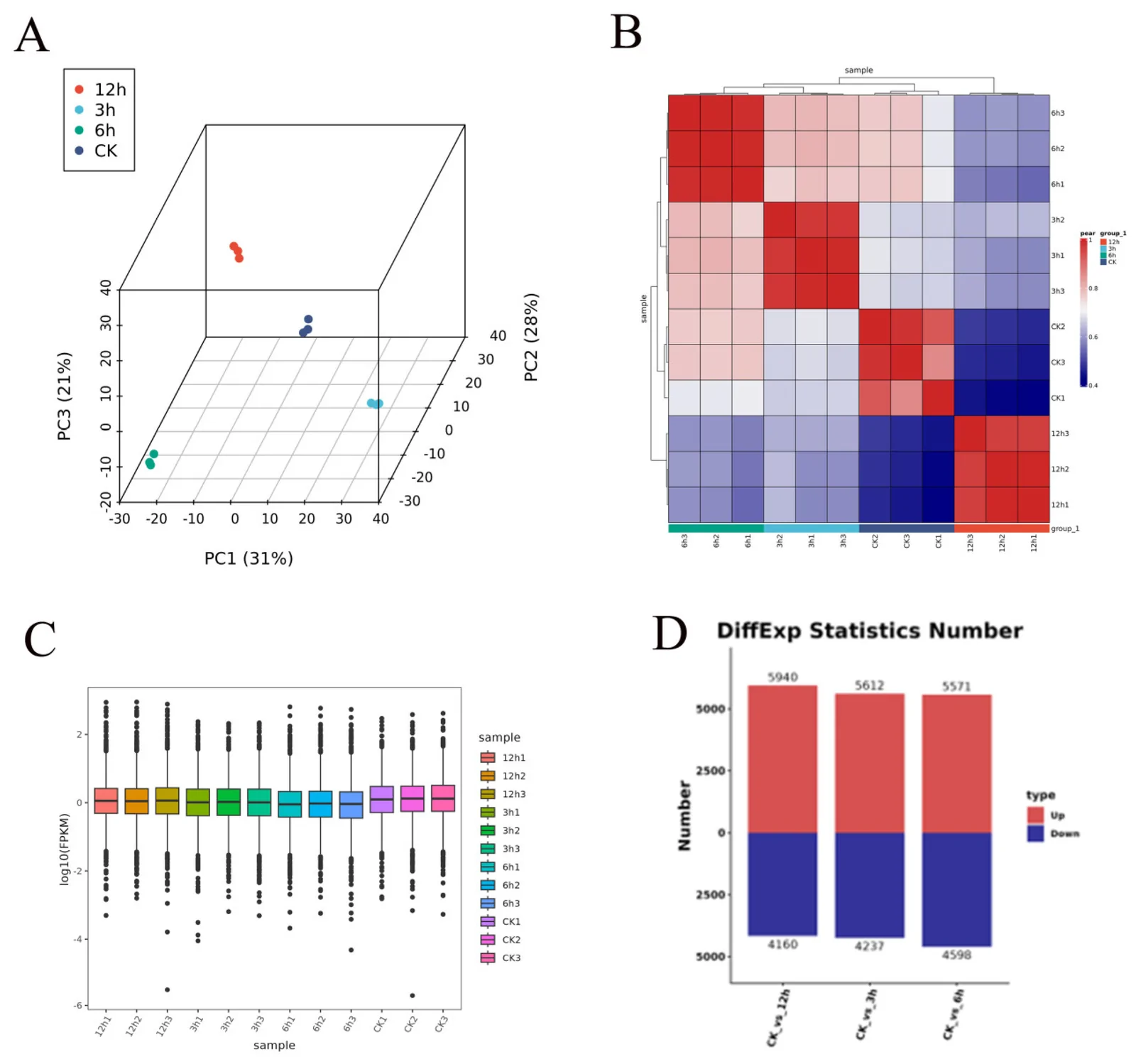
(**A**) Principal component analysis (PCA), (**B**) sample correlation analysis, (**C**) boxplot visualization, and (**D**) DEG statistics across different sample groups.

To elucidate transcriptional changes in leaves under freezing stress (−4 °C), differential expression analysis was performed using the cultivar DX27 as the experimental material, with normal temperature (25 °C) as the control (CK). Correlation analysis and principal component analysis (PCA) were conducted to simplify transcriptome data complexity and evaluate repeatability among biological replicates. Samples from identical treatments were clearly clustered, and the largest separation between the control and stressed groups in the PCA plot was detected at 12 h (Figure 1A). The correlation coefficients for the three replicates of the four samples were all close to 1, indicating the high similarity of expression patterns among the samples (Figure 1B). Boxplot visualization of the data demonstrated consistency among samples within the same treatment group, confirming the reliability of the biological replicates (Figure 1C). 10,100 DEGs (5940 up-regulated, 4160 down-regulated), 9849 DEGs (5612 up-regulated, 4488 down-regulated), and 10,169 DEGs (5571 up-regulated, 4598 down-regulated) were detected at CK-vs-3h, CK-vs-6h, and CK-vs-12h (Figure 1D and Supplementary Figure S1) with the threshold set at |log_2_FC| ≥ 1 and Padj < 0.05. Venn-diagram analysis was performed to dissect shared and stage-specific DEGs across three freezing-stress time points. A total of 2206, 2080 and 2774 non-redundant DEGs were obtained for CK-vs-3h, CK-vs-6h and CK-vs-12h, respectively. Among them, 4252 DEGs were shared by all three time-points, while a considerable number of genes exhibited time-point-specific or two-group-shared expression patterns. Collectively, 6161 unique DEGs were detected in at least one of the three comparisons (Supplementary Figure S2).

### 3.2. Functional and Pathway Enrichment Analysis of DEGs Under Freezing Stress

**Figure 2:**
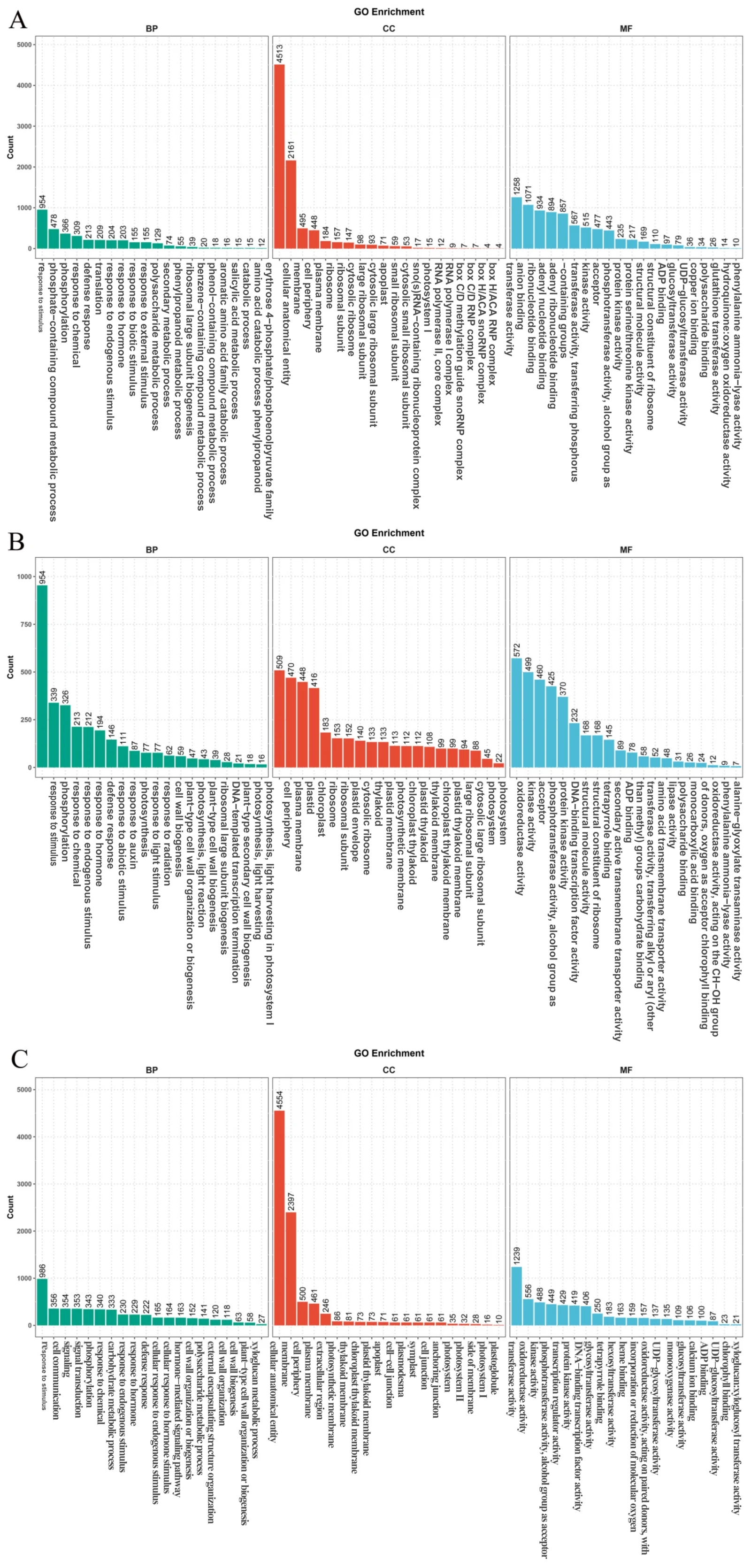
Gene Ontology (GO) enrichment analysis of DEGs in pea leaves under freezing stress (−4 °C). (**A**) GO enrichment analysis of DEGs in CK_vs_3h; (**B**) GO enrichment analysis of DEGs in CK_vs_6h; (**C**) GO enrichment analysis of DEGs in CK_vs_12h.

GO enrichment analysis classified the DEGs into three primary categories—Biological Process (BP), Cellular Component (CC), and Molecular Function (MF)—revealing shared functional characteristics and time-dependent transcriptional changes across the three sampling time points (Figure 2). In the CK_vs_3h comparison, the top enriched Cellular Component terms were Cytosolic ribosome (GO:0022626), Ribosomal subunit (GO:0044391), and Cytosolic large ribosomal subunit (GO:0022625). The Molecular Function domain showed enrichment in the following categories: Protein kinase activity (GO:0004672), Phosphotransferase activity, alcohol group as acceptor (GO:0016773), and Kinase activity (GO:0016301). The Biological Process domain revealed the following top enriched terms: Phosphorylation (GO:0016310), Defense response (GO:0006952), and Response to stimulus (GO:0050896) (Figure 2A). In CK_vs_6h, the top enriched terms in the Cellular Component (CC) category were chloroplast (GO:0009507), plastid (GO:0009536), ribosome (GO:0005840), and thylakoid (GO:0009579); in the Molecular Function (MF) domain, the most prominently enriched terms were protein kinase activity (GO:0004672), structural constituent of ribosome (GO:0003735), and oxidoreductase activity (GO:0016491); in the Biological Process (BP) category, the most enriched terms were photosynthesis (GO:0015979), response to stimulus (GO:0050896), defense response (GO:0006952), and response to chemical (GO:0042221) (Figure 2B). In CK_vs_12h, the top enriched terms in the Cellular Component (CC) category were membrane (GO:0016020), plasma membrane (GO:0005886), and cell peripher (GO:0071944); the Molecular Function (MF) domain, the most enriched terms were DNA-binding transcription factor activity (GO:0003700), protein kinase activity (GO:0004672), and tetrapyrrole binding (GO:0046906); in the Biological Process (BP) category the top enriched terms were response to stimulus (GO:0050896), followed by defense response (GO:0006952) and response to chemical (GO:0042221) (Figure 2C).

**Figure 3:**
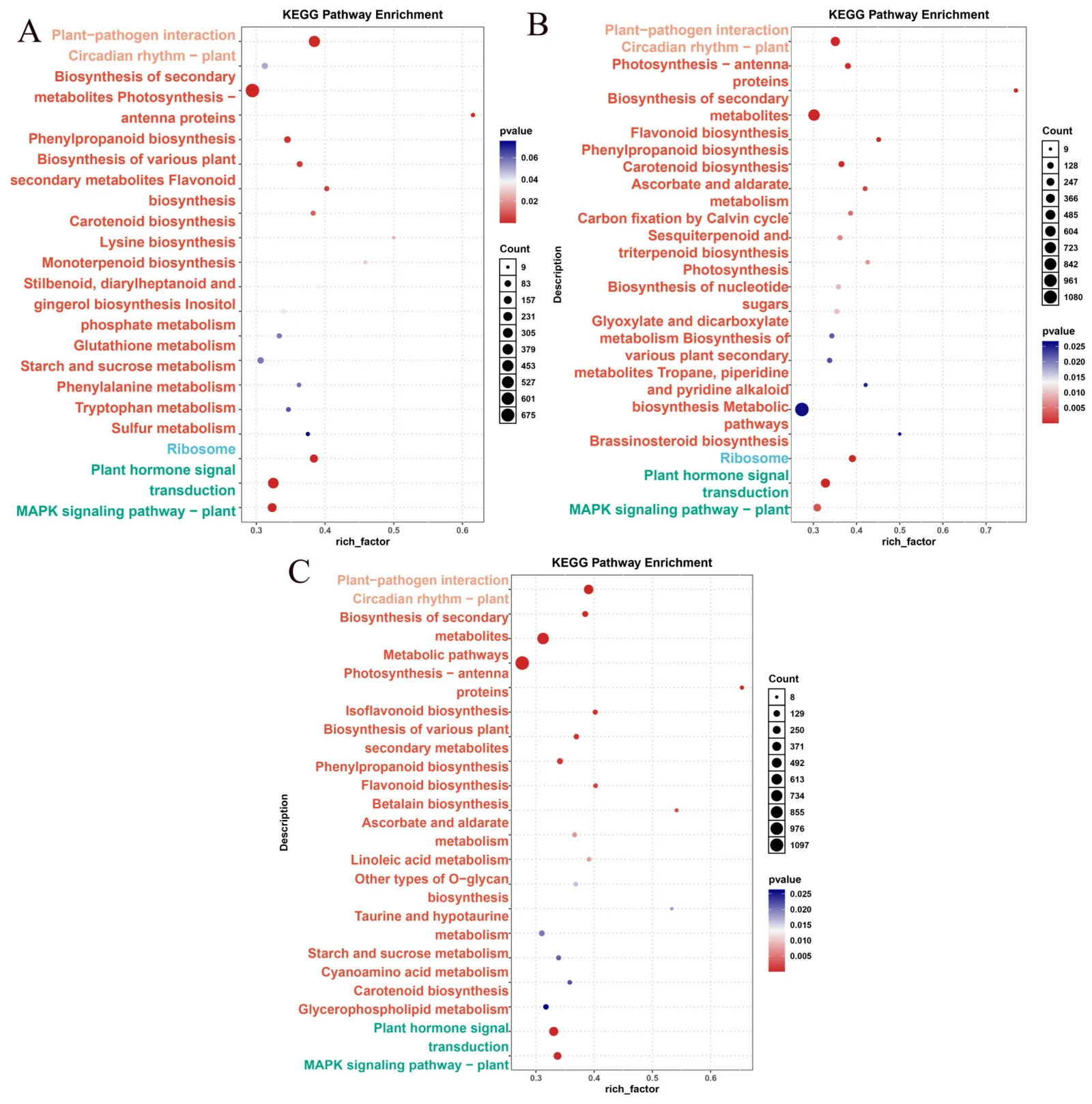
KEGG pathway enrichment analysis of DEGs in pea leaves under freezing stress for 3, 6, and 12 h. (**A**) KEGG pathway enrichment analysis of DEGs in CK_vs_3h; (**B**) KEGG pathway enrichment analysis of DEGs in CK_vs_6h; (**C**) KEGG pathway enrichment analysis of DEGs in CK_vs_12h.

KEGG pathway enrichment analysis was performed on the differentially expressed genes. In this section, we compared cold-stressed samples with CK to dissect the molecular responses under freezing stress(Figure 3). In the CK_vs_3h comparison, Biosynthesis of secondary metabolites (ko01110), Glutathione metabolism (ko00480), Plant hormone signal transduction (ko04075), MAPK signaling pathway—plant (ko04016), and Ribosome (ko03010) pathway were significantly up-regulated, as indicated by positive z-scores. RNA degradation (ko03018) and Spliceosome (ko03040) were enriched with negative z-scores, suggesting down-regulation. In the CK_vs_6h comparison, the Biosynthesis of secondary metabolites (ko01110), Plant hormone signal transduction (ko04075), MAPK signaling pathway—plant (ko04016), and Ribosome (ko03010) pathways were significantly up-regulated, as indicated by strong positive z-scores (4.95, 5.38, 2.87, and 6.21, respectively). Conversely, several pathways involved in RNA processing and protein trafficking were enriched with negative z-scores, suggesting down-regulation. These included Spliceosome (ko03040), Nucleocytoplasmic transport (ko03013), mRNA surveillance pathway (ko03015), Protein processing in endoplasmic reticulum (ko04141), Endocytosis (ko04144), and RNA degradation (ko03018). In the CK_vs_12h comparison, the Biosynthesis of secondary metabolites (ko01110), Plant hormone signal transduction (ko04075), and MAPK signaling pathway—plant (ko04016) pathways remained significantly up-regulated. In striking contrast, the Ribosome (ko03010) pathway, which was up-regulated at 3 h and 6 h, exhibited a strong negative z-score of −8.23 at 12 h, indicating a pronounced down-regulation of translation. Conversely, several pathways involved in RNA processing and protein trafficking continued to show negative z-scores, suggesting sustained down-regulation. These included Spliceosome (ko03040), Nucleocytoplasmic transport (ko03013), mRNA surveillance pathway (ko03015), Protein processing in endoplasmic reticulum (ko04141), Endocytosis (ko04144), and RNA degradation. Glutathione metabolism, identified as a major pathway involved in cold stress response, warrants further investigation.

### 3.3. Identification of the Pea GST Gene Family

A total of 52 members of the *GST* gene family were identified from the pea genome using the hidden Markov model (HMM) profiles corresponding to the conserved GST domains (GST-N: PF02798; GST-C: PF00043). All identified proteins contained complete GST-N or GST-C conserved domains. Phylogenetic analysis further classified these 52 PsatGST genes into nine distinct subfamilies, which were then systematically designated.

### 3.4. Phylogenetic Analysis of the Pea PsatGST Gene Family

**Figure 4:**
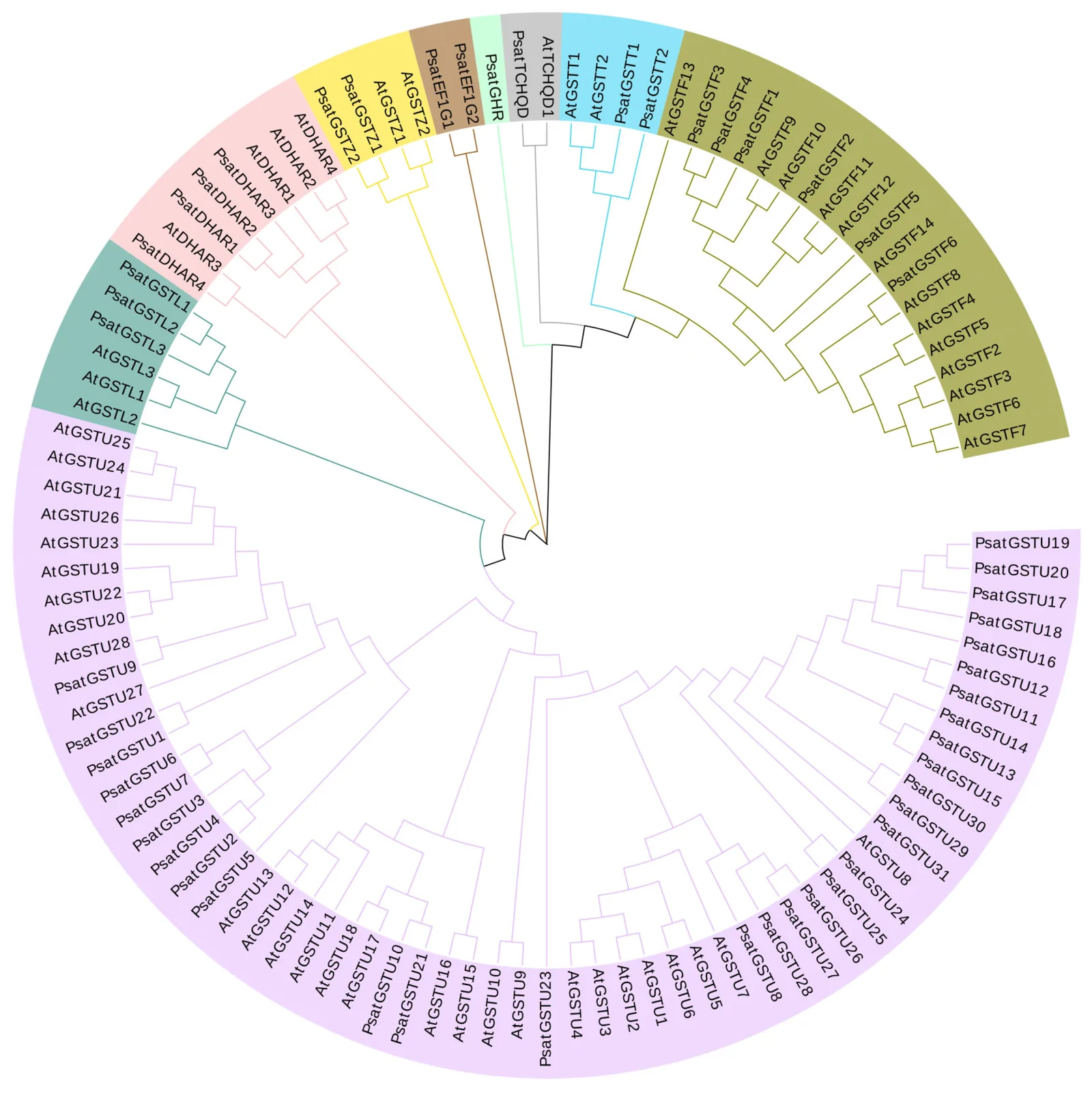
Phylogenetic tree of the pea and *Arabidopsis thaliana GST* gene family. Each color corresponds to a distinct gene subfamily.

To clarify the phylogenetic relationships among the 52 identified PsatGST proteins, multiple sequence alignment was carried out, followed by construction of a neighbor-joining phylogenetic tree with 1000 bootstrap replications. As depicted in Figure 4, these *PsatGST* genes were divided into nine distinct subfamilies. The Tau subfamily was the largest, comprising 31 members (accounting for 59.6% of the total), followed by the Phi subfamily with six members (11.5%). The DHAR subfamily contained four members (7.7%), while the Lambda subfamily contained three members (5.8%), and the Zeta, Theta, and EF1G subfamilies each contained two members (3.9%). The GHR and TCHQD branches each contained only one member (1.9%). This subfamily distribution pattern is consistent with observations in other plant species, where the Tau and Phi classes typically constitute the largest subfamilies.

### 3.5. Conserved Motif and Gene Structure Analysis of the PsatGST Gene Family

**Figure 5:**
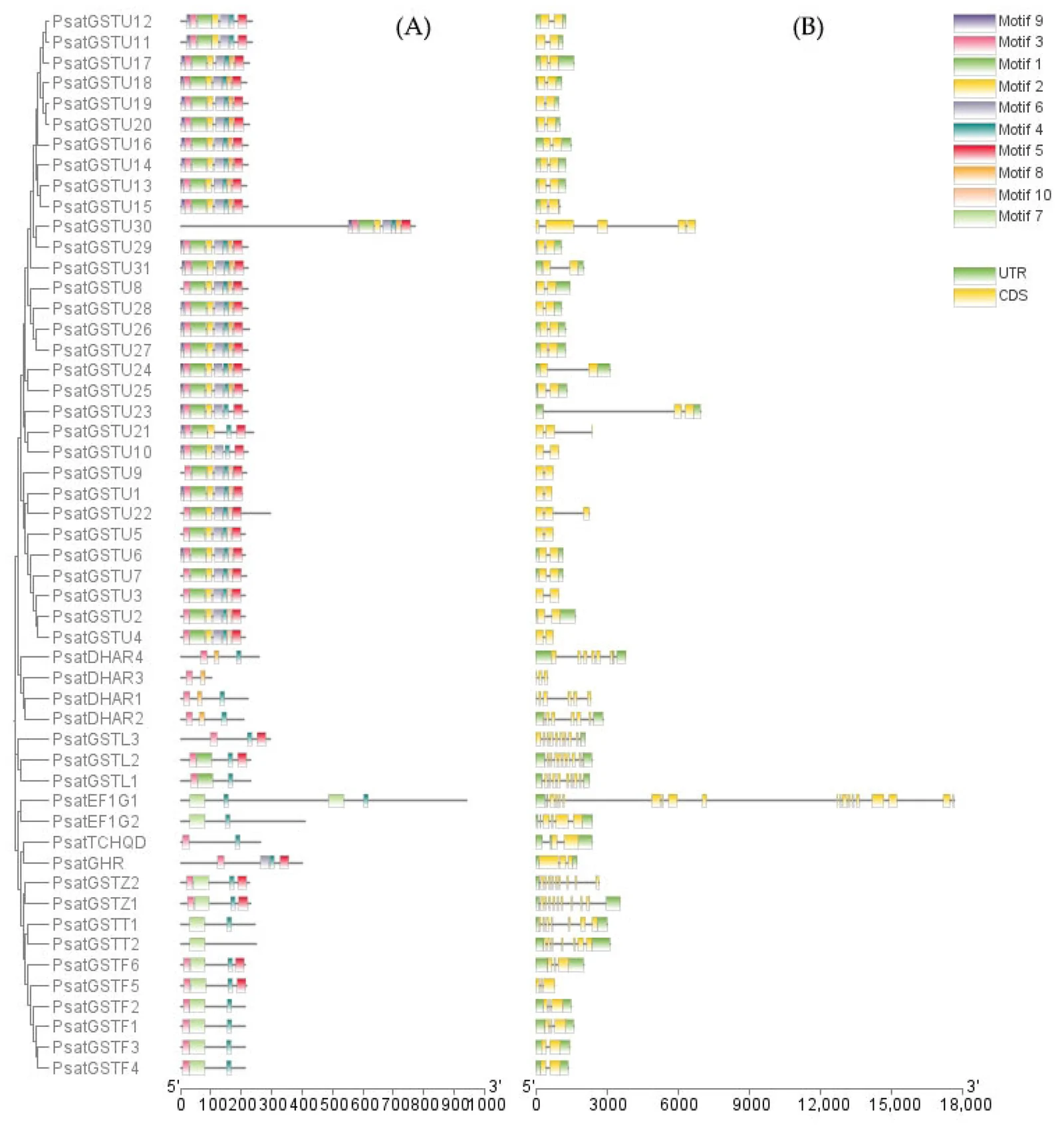
(**A**) Conserved motifs and (**B**) gene structures of *PsatGST* genes.

To investigate the structural diversification of the *PsatGST* gene family in peas, comprehensive comparative analyses were conducted on the 52 *PsatGST* sequences to characterize conserved motifs and gene structures. As illustrated in Figure 5, the MEME suite identified 10 conserved motifs across all family members. Most *PsatGSTs* harbored 3 to 6 conserved motifs, and motif 3 was detected in nearly all proteins, indicating a high degree of evolutionary conservation, although its exact biochemical role remains to be verified experimentally. The Tau, Phi, and Lambda subfamilies shared an identical arrangement of motif 1, motif 3, motif 4, and motif 5, implying conserved structural features within these clades. Notably, the Phi subfamily uniquely possessed motif 7, which may contribute to substrate specificity or subfamily-specific functional specialization. The conserved motif distribution within each subfamily suggests that these motifs are critical to the physiological functions of their corresponding PsatGST proteins.

Gene structure analysis revealed that all 52 *PsatGST* gene family members contained introns, indicating that pea *PsatGST* family members may have undergone multiple evolutionary trajectories with certain subfamily-specific features. Among them, Lambda subfamily members had the highest number of introns (nine introns each). Tau subfamily members consistently contained only a single relatively short intron, which might hypothetically enable more rapid transcriptional activation under stress, but this remains to be tested experimentally. Phi subfamily members generally contained two introns, while other family members exhibited 2–8 introns. The distinct intron–exon patterns among subfamilies reflect their evolutionary divergence and potential functional specialization.

### 3.6. Chromosomal Localization of the PsatGST Gene Family

**Figure 6:**
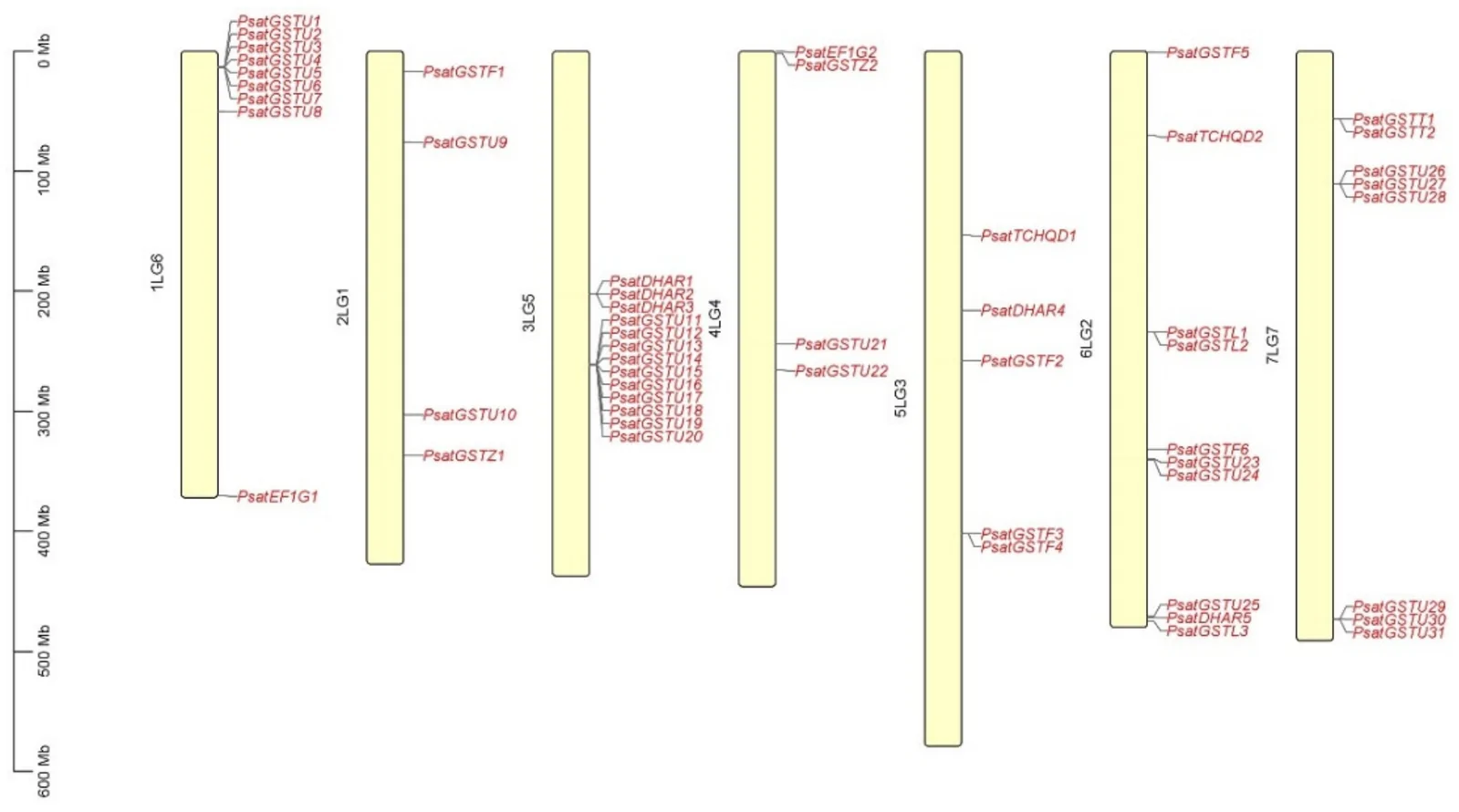
Chromosomal distribution of pea *PsatGST* genes.

Chromosomal mapping analysis showed that *PsatGST* genes are unevenly distributed across the pea genome. A total of 52 *PsatGST* gene family members were mapped onto seven assembled chromosomes (Figure 6). Among these, chromosome 3LG5 harbored the highest number of genes, forming a high-density gene cluster. The lowest numbers of genes were observed in chromosomes 2LG1 and 4LG4, with only four genes mapped.

### 3.7. Promoter Cis-Element Analysis of the PsatGST Gene Family

**Figure 7:**
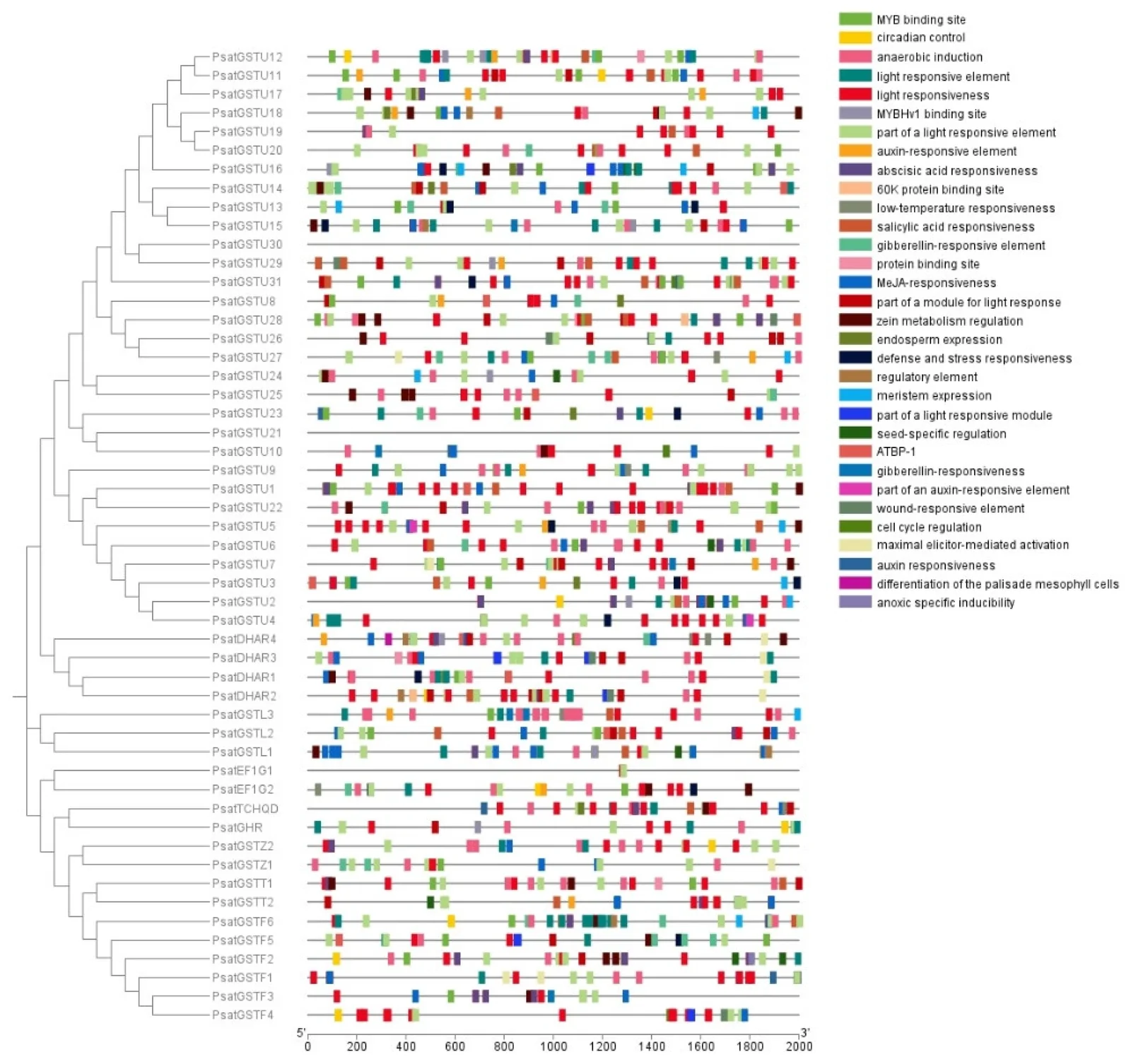
Cis-acting regulatory elements in the promoter regions of pea *PsatGST* genes. The 2000 bp upstream sequences of each *PsatGST* gene were retrieved and analyzed for cis-element identification, with different colored boxes corresponding to distinct types of cis-acting elements.

To explore potential regulatory clues underlying *PsatGST* transcription, the 2000 bp upstream of the translation initiation site (ATG) of each *PsatGST* gene were extracted and subjected to cis-element prediction via the online PlantCARE database. Identified cis-acting elements were visualized using the TBtools software package. As shown in Figure 7, numerous regulatory motifs associated with plant growth and development, hormone signal transduction, and stress responses were broadly distributed across these promoter regions. The widespread occurrence of these light-related cis-elements only reveals a latent regulatory link to light signal cascades, while the direct participation of *PsatGSTs* in light signaling pathways requires further experimental validation. Furthermore, multiple stress-associated motifs were also identified, including the anaerobic induction element (ARE), low temperature responsive element (LTR), wound-responsive WUN motif, and TC-rich repeats linked to defense responses. The presence of these stress-related cis-elements merely suggests that *PsatGST* genes may receive diverse abiotic and biotic stress signals at the transcriptional level. It is not possible to determine the definite biological roles of *PsatGSTs* in stress adaptation based solely on promoter motif distribution.

### 3.8. Expression Pattern Analysis of the Pea PsatGST Gene Family Under Freezing Stress

**Figure 8:**
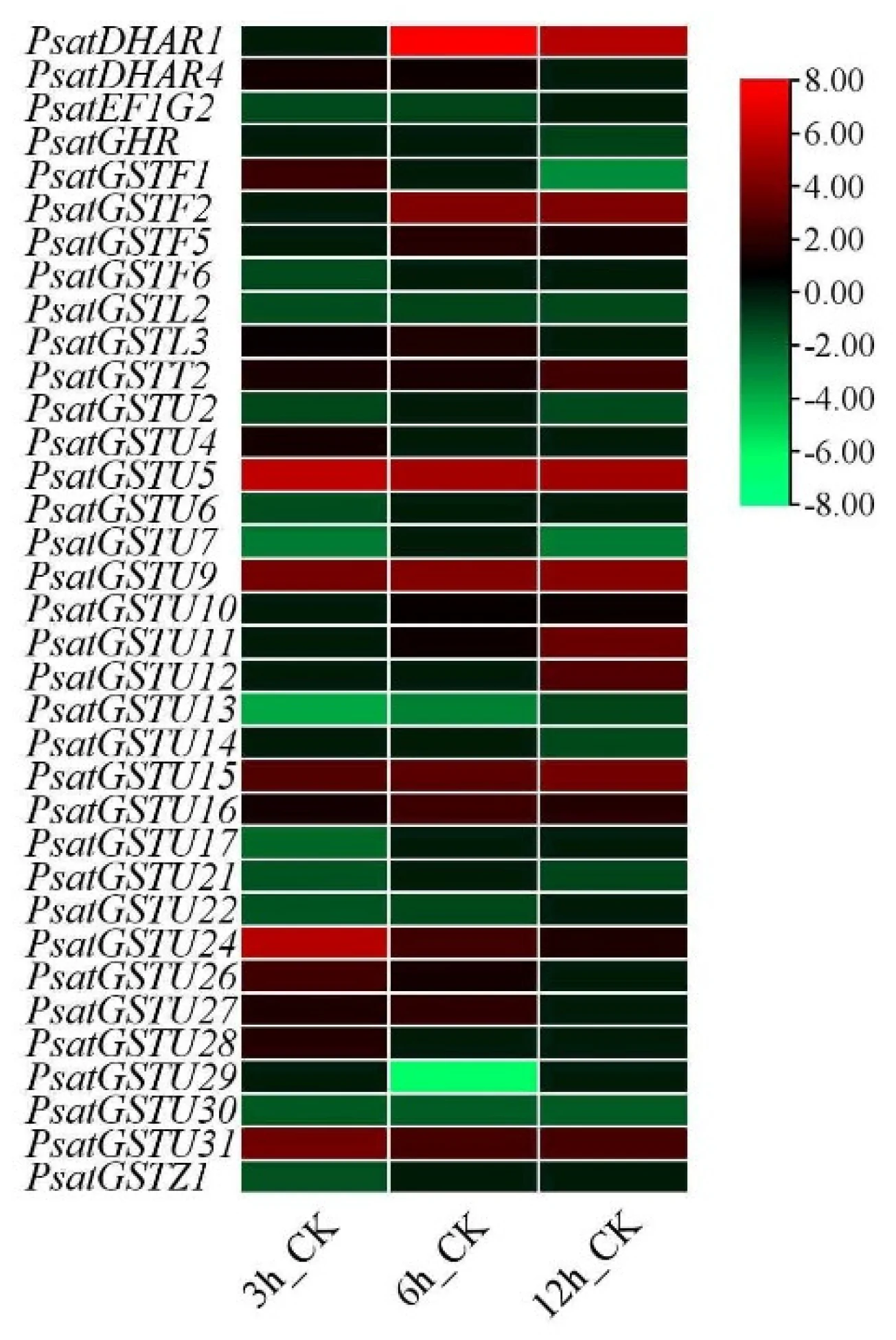
Expression profiling of *PsatGST* genes in leaves under freezing stress. Heatmap showing log_2_-transformed values of *PsatGST* genes at 3 h, 6 h, and 12 h under control (25 °C) and freezing stress (−4 °C) conditions. Color scale represents relative expression levels from low (green) to high (red).

To explore the transcriptional responses of *PsatGST* genes to freezing stress, their expression profiles were analyzed using transcriptome sequencing data. The sequencing was performed at 3 h, 6 h, and 12 h under freezing treatment (−4 °C), with samples cultured at 25 °C set as the CK. Genes with raw counts <10 in all samples were defined as non-expressed genes; accordingly, only 31 *PsatGST* genes were expressed in the transcriptome, and 21 genes were not expressed. Hierarchical clustering revealed distinct expression dynamics among different subfamilies and individual genes (Figure 8). Several genes, including *PsatDHAR1*, *PsatGSTF2*, *PsatGSTU5*, *PsatGSTU9*, and *PsatGSTU15* were significantly and persistently up-regulated under freezing stress. In sharp contrast, *PsatGSTL2*, *PsatGSTU13*, *PsatGSTU22*, and *PsatGSTU33* were significantly and persistently down-regulated under freezing stress. *PsatGSTU29* was down-regulated at 6 h under freezing stress. *PsatGSTF1* was down-regulated at 12 h under freezing stress.

**Figure 9:**
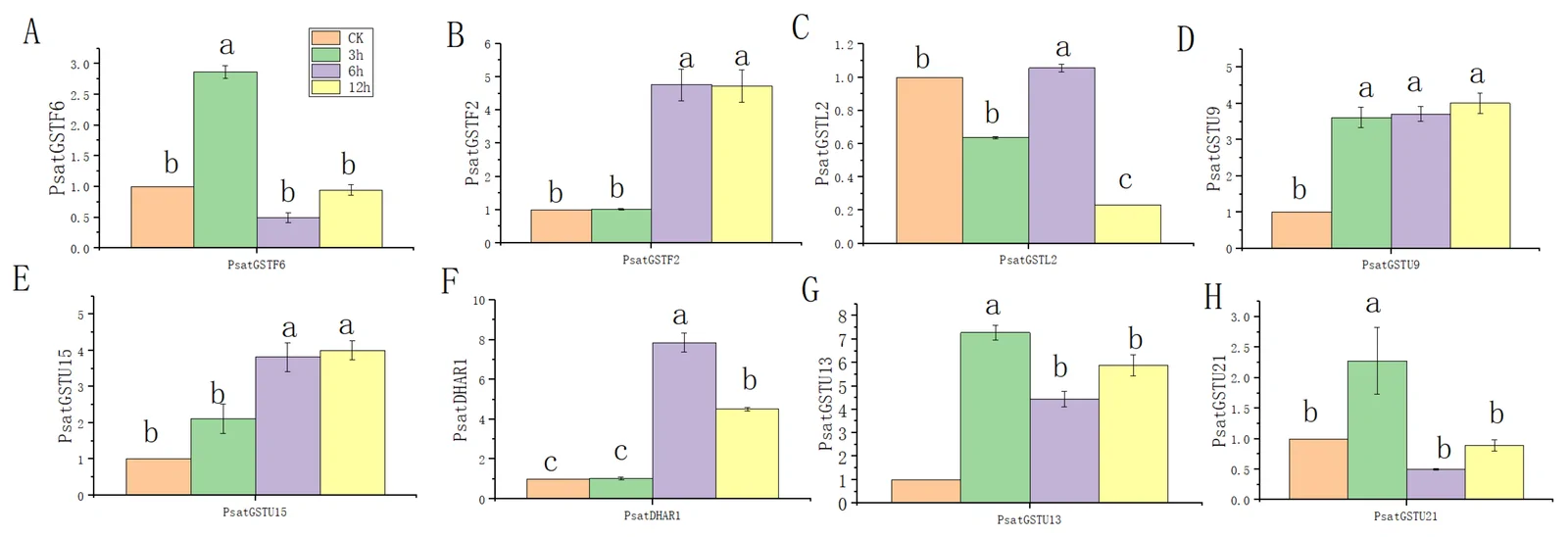
(**A**) The expression level of *PsatGSTF6* gene under different stress treatments. (**B**) The expression level of *PsatGSTF2* gene under different stress treatments. (**C**) The expression level of *PsatGSTL2* gene under different stress treatments. (**D**) The expression level of *PsatGSTU9* gene under different stress treatments. (**E**) The expression level of *PsatGSTU15* gene under different stress treatments. (**F**) The expression level of *PsatDHAR1* gene under different stress treatments. (**G**) The expression level of *PsatGSTU13* gene under different stress treatments. (**H**) The expression level of *PsatGSTU21* gene under different stress treatments. Different lowercase letters represent significant differences in freezing treatment times (*p* < 0.05). The error bar represents the standard deviation of the average of the sample.

To comprehensively characterize the expression patterns of pea glutathione S-transferase-family genes under freezing stress, quantitative real-time PCR (qRT-PCR) was used to measure the relative transcript levels of eight *PsatGST* genes in pea leaves. Eight *PsatGST* genes were selected for further analysis because they represent the main GST subfamilies (Tau, Phi, Lambda, DHAR, etc.) identified in the pea genome, exhibited significant differential expression (|log_2_FC| ≥ 1, FDR < 0.05) in at least one of the three freezing-stress time points according to our RNA-seq data, and comprised both strongly up-regulated and representative moderately modulated genes across time points. (Figure 9) The genes based on distinct expression patterns and subfamily representation from RNA-seq data. The eight tested *PsatGST* genes displayed diverse expression changes under freezing stress.

## 4. Discussion

Transcriptomic profiling revealed functional differentiation and stage-specific transcriptional patterns of the *PsatGST* gene family in the leaves of the pea cultivar DX27 cultivar under freezing stress. GO enrichment analysis showed highly consistent functional enrichment categories across all treatment groups, dominated by oxidation–reduction processes, stress responses, membrane-related biological functions, and catalytic activities. This overall enrichment profile shares a high degree of similarity with the canonical biological functions of GST proteins reported in previous research [23]. Venn-diagram comparisons of DEGs across 3 h, 6 h and 12 h freezing treatments illustrated that pea freezing-stress response relied on both a large persistent core gene set and progressive recruitment of additional stage-specific genes. A substantial proportion of DEGs (4252 genes) were consistently differentially expressed throughout the whole freezing treatment, implying their fundamental roles in sustaining basal stress adaptation. Meanwhile, substantial numbers of genes were uniquely activated or repressed at individual time-points or shared between two time-points, indicating that distinct transcriptional programmes are sequentially triggered as freezing injury accumulates. Continuous enrichment of oxidation–reduction processes in the BP category and oxidoreductase activity in the MF category at all sampling time points provides only the transcriptomic background associated with GST-mediated redox homeostasis, rather than definitive functional evidence for individual *PsatGST* genes.

In this study, a total of 52 *PsatGST* members were identified and classified into nine distinct subfamilies. Genes within the same subfamily shared conserved gene structures and protein motifs, and the majority of members belonged to Phi and Tau subfamilies, two subgroups frequently reported to participate in plant abiotic stress responses in other plant species. Minor structural divergence across different subfamilies may underlie potential functional differentiation of the pea *GST* family. The uneven distribution of *PsatGST* genes across seven pea pseudochromosomes is consistent with the evolutionary characteristics of *GST* families in other higher plants [35,36,37]. A limitation of this study is that a comprehensive tandem and segmental duplication analysis of the *PsatGST* gene family was not performed; such analysis would further clarify the contribution of whole-genome and tandem duplication events to GST family expansion in pea. Future work incorporating synteny analysis will be needed to fully resolve the evolutionary dynamics of this gene family.

Promoter cis-element analysis provides preliminary clues about the transcriptional regulation of *PsatGST* genes. The widespread presence of light-responsive motifs (G-box, GT1-motif, I-box) in promoter regions indicates only a potential regulatory linkage between light signaling and cold response pathways. Previous studies have proven that light can modulate plant stress tolerance by regulating antioxidant systems in other crops [35,36,37]. The frequent occurrence of MYB-binding sites in *PsatGST* promoters corresponds to published evidence that MYB transcription factors mediate the expression of numerous stress-responsive genes under cold and drought conditions [38]. Direct regulatory relationships between these cis-motifs and the cold-induced transcription of *PsatGSTs* require further experimental verification. DHAR represents a special subgroup within the GST superfamily and is a key enzyme of the AsA-GSH cycle responsible for ascorbate regeneration. [39,40]. The sustained up-regulation of *PsatDHAR1* and *PsatDHAR4* under cold treatment implies that the AsA-GSH cycle may be continuously activated to regenerate ascorbate, which contributes to ROS scavenging and alleviates cold-triggered oxidative damage.

Consistent correlations between GST expression and cold adaptation have been observed across various plant species. Thirty-nine *CmGST* genes were identified in melon (*Cucumis melo*); cold-adapted melon varieties exhibited higher GST enzyme activity and up-regulated expression of 28 stress-responsive *GST* genes mainly from the Tau, Phi, and DHAR subfamilies [41]. In walnuts (*Juglans regia*), transgenic plants overexpressing *JrGSTU1* displayed significantly higher GST enzyme activity relative to wild-type and RNAi lines, showing that elevated GST abundance can enhance cold tolerance in heterologous systems [42]. In winter rapeseed (*Brassica napus*), multiple *GST* genes (including *BnaA02g35760D*, *BnaC06g20450D*, *BnaC06g35490D*) were markedly induced under freezing conditions in cold-adapted accessions, with stably expressed genes (*BnaA03g26130D*, *BnaC03g30880D*) and transiently induced genes (*BnaC01g39170D*, *BnaC08g37940D*) showing distinct expression trends during cold acclimation [43]. Cold stress strongly induced *BraGSTF2* in winter turnip rape plants, further supporting that Phi-subfamily *GSTs* are commonly linked to cold response processes across plant taxa [35].

The Tau subfamily constitutes the largest subgroup of pea *PsatGSTs*, containing 31 members (60.8%) with highly diversified expression patterns; these include constitutive high expression or low basal expression. Such broad transcriptional heterogeneity suggests the Tau subfamily makes major contributions to the cold-triggered transcriptional reprogramming of DX27, reflecting the evolutionary plasticity of the *PsatGST* family. The divergent temporal expression profiles of *PsatGSTU27* and *PsatGSTU4* suggest possible coordinated transcriptional activation among Tau-subfamily members under freezing treatment.

RNA-seq was used to characterize transcriptional changes in the *PsatGST* family in the moderately cold-tolerant pea cultivar DX27 under freezing stress. Multiple genes, including *PsatDHAR1*, *PsatGSTF2*, *PsatGSTU5*, *PsatGSTU9*, and *PsatGSTU15,* were significantly up-regulated under freezing treatment compared with the control group. The following set of genes with sustained expression during exposure to freezing conditions was screened: *PsatDHAR1, PsatGSTF2*, *PsatGSTU5*, *PsatGSTU9*, *PsatGSTU15*, *PsatGSTL2*, *PsatGSTU13*, *PsatGSTU22*, *PsatGSTU33*, *PsatGSTU29*, and *PsatGSTF1*. Collectively, transcriptome and qRT-PCR results only identify a panel of cold-associated *PsatGST* candidate genes with altered expression correlations under freezing conditions. In addition, some *PsatGSTs* exhibited opposite expression trends under freezing stress, suggesting that different genes respond to distinct types of stress. Although candidate genes including *PsatDHAR1*, *PsatGSTF2*, and Tau-class GSTs were identified based on transcriptomic data, functional validation experiments, such as overexpression, knockout/knockdown, and enzyme activity assays, have not been carried out. Therefore, further investigations are required to confirm their biological functions in pea freezing stress response. Conversely, other genes showed similar expression patterns under both stress conditions, reflecting both the functional specificity and pleiotropy of *GST* genes [35]. This study provides comprehensive insights into pea freezing-stress responses at the transcript level based on *PsatGSTs* expression profiles, and offers novel clues to uncover the molecular mechanisms of pea freezing tolerance.

## Figures and Tables

**Table 1:** Gene-specific primers of *PsatGST* genes and reference gene used for qRT-PCR.

Genes	Primer
*EF1α-F*	CTGTGCTTATTGTCGCTGCT
*EF1α-R*	TCGCTGTATGGTGGTTCAGT
*PsatGSTL2-F*	TTTGAATACTTGGAGAATGCCCTTGG
*PsatGSTL2-R*	TCAACGAACGGAACATACGCAATATC
*PsatDHAR1-F*	TGAGCCATTCTTTGTTACTCCTCCTC
*PsatDHAR1-R*	AAGCATTCAGTTCAGCAACCAAGG
*PsatGSTF6-F*	CACATTGGTGGATCTGCATCACTTAC
*PsatGSTF6-R*	CAGCAACCCAAGCACTAACATGAG
*PsatGSTF2-F*	AGAGAGTGTTGGCTTGCCTCATAG
*PsatGSTF2-R*	TCACCGTCCTCTATTACTGGAACTTG
*PsatGSTU21-F*	TGATAGAGTTCTTGCTCGGTTCTGG
*PsatGSTU21-R*	CTCCATCCTCTCAACCACTTCTTCC
*PsatGSTU13-F*	CCCGATACTGCCTTCTGATCCTTAC
*PsatGSTU13-R*	CCTCAATACCTTGCTCAAGCTCTTTC
*PsatGSTU5-F*	TGGGCTAACTACGTTGACACTAAGG
*PsatGSTU5-R*	GCACCTTCTTTCTCTTCTCCTTCAC
*PsatGSTU9-F*	AATGGAGAAGAGAGAGAAGTGGGAAC
*PsatGSTU9-R*	GCAGAATGAGGAATGGCTATGATGTC

## Data Availability

The genome sequence of *P. sativum* was retrieved from public databases. All datasets generated and analyzed during this study are available from the corresponding author upon reasonable request.

## Supplementary Materials

The following supporting information can be downloaded at: https://www.mdpi.com/article/10.3390/genes17091052/s1, Figure S1: Volcano plots of DEGs under freezing stress at three time points. Volcano plots showing the distribution of gene expression changes for comparisons of stress-treated seedlings versus time-matched controls. (A) CK-vs-3h; (B) CK-vs-6h; (C) CK-vs-12h. The *x*-axis represents log_2_(fold change), and the *y*-axis represents −log_10_(adjusted *p*-value). Red dots indicate significantly up-regulated genes, blue dots indicate significantly down-regulated genes, and gray dots represent genes with no significant expression difference. The numbers in parentheses denote the gene counts for each category. Figure S2: Venn-diagram analysis of differentially expressed genes (DEGs) identified from three freezing-stress comparisons. Venn diagram showing the overlaps of DEGs identified in CK-vs-3h, CK-vs-6h and CK-vs-12h comparisons under freezing stress. Each circle represents the total number of non-redundant DEGs detected in each time-point comparison. Overlapping regions indicate shared DEGs among different groups. In total, 6161 unique DEGs were differentially expressed in at least one time-point. Table S1: Gene expression at different time points.
