# Supplementary material for: Genome-Wide Identification of the PsatGST Gene Family and Expression Analysis Under Freezing Stress in Peas (Pisum sativum L.)
Source: Genes (Basel). 2026 Aug 31;17(9):1052. doi: 10.3390/genes17091052 (PMC13606755; doi:10.3390/genes17091052)
Supplement: Supplementary file 1 [file genes-17-01052-s001.zip › genes-4483840-supplementary Figure.pdf]

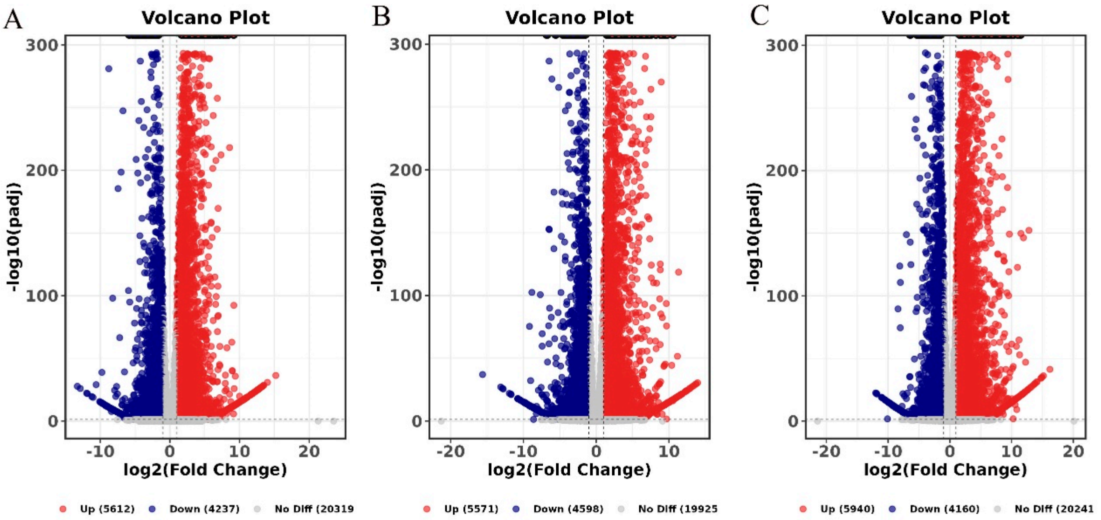

Figure S1. Volcano plots of DEGs under freezing stress at three time points. Volcano plots showing the distribution of gene expression changes for comparisons of stress-treated seedlings versus time-matched controls. (A) CK-vs-3 h; (B) CK-vs-6 h; (C) CK-vs-12 h. The x-axis represents $\log_2$(fold change), and the y-axis represents $-\log_{10}$(adjusted P-value). Red dots indicate significantly up-regulated genes, blue dots indicate significantly down-regulated genes, and gray dots represent genes with no significant expression difference. The numbers in parentheses denote the gene counts for each category.

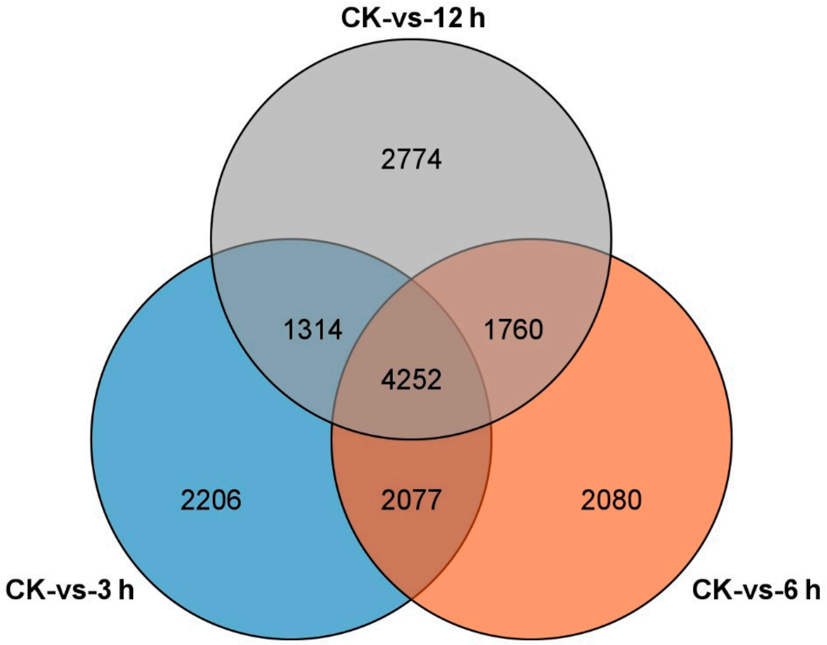

Supplementary Figure S2. Venn-diagram analysis of differentially expressed genes (DEGs)

identified from three freezing-stress comparisons.Venn diagram showing the overlaps of DEGs identified in CK-vs-3 h, CK-vs-6 h and CK-vs-12 h comparisons under freezing stress. Each circle represents the total number of non-redundant DEGs detected in each time-point comparison. Overlapping regions indicate shared DEGs among different groups. In total, 6161 unique DEGs were differentially expressed in at least one time-point.
